# Synaptic Mechanisms of Ethanol Tolerance and Neuroplasticity: Insights from Invertebrate Models

**DOI:** 10.3390/ijms25136838

**Published:** 2024-06-21

**Authors:** Aakriti Bhandari, Alexandra Seguin, Adrian Rothenfluh

**Affiliations:** 1 Department of Psychiatry, University of Utah, Salt Lake City, UT 84112, USA; 2 Molecular Medicine Program, University of Utah, Salt Lake City, UT 84112, USA; 3 Neuroscience Graduate Program, University of Utah, Salt Lake City, UT 84112, USA; 4 Department of Neurobiology, University of Utah, Salt Lake City, UT 84112, USA; 5 Department of Human Genetics, University of Utah, Salt Lake City, UT 84112, USA

**Keywords:** *Drosophila*, *C. elegans*, invertebrates, alcohol tolerance, neuroplasticity, AUD, neurotransmission

## Abstract

Alcohol tolerance is a neuroadaptive response that leads to a reduction in the effects of alcohol caused by previous exposure. Tolerance plays a critical role in the development of alcohol use disorder (AUD) because it leads to the escalation of drinking and dependence. Understanding the molecular mechanisms underlying alcohol tolerance is therefore important for the development of effective therapeutics and for understanding addiction in general. This review explores the molecular basis of alcohol tolerance in invertebrate models, *Drosophila* and *C. elegans*, focusing on synaptic transmission. Both organisms exhibit biphasic responses to ethanol and develop tolerance similar to that of mammals. Furthermore, the availability of several genetic tools makes them a great candidate to study the molecular basis of ethanol response. Studies in invertebrate models show that tolerance involves conserved changes in the neurotransmitter systems, ion channels, and synaptic proteins. These neuroadaptive changes lead to a change in neuronal excitability, most likely to compensate for the enhanced inhibition by ethanol.

## 1. Introduction

Alcohol use disorder (AUD) is a complex disorder impacting millions of individuals worldwide. It includes the compulsive and excessive consumption of alcohol despite its detrimental effects on physical and mental well-being. In the 2021 national drug and health surveys, approximately 29.5 million individuals aged 12 and older in the United States alone reported having experienced AUD in the previous year [1]. The global pandemic of COVID-19 has increased the prevalence of this disorder because increased stress, social isolation, and economic hardship contribute to the development and escalation of AUD [2]. The consequences of AUD extend across a spectrum of physical, psychological, and social issues, including liver disease [3,4,5,6], cardiovascular problems [5,7,8,9], strained interpersonal relationships, and cognitive impairments [10,11,12]. Therefore, gaining a comprehensive understanding of its underlying mechanisms is crucial to identifying not only at-risk individuals but also developing more efficient therapeutics.

The development and progression of AUD are significantly influenced by tolerance, a neuroadaptive mechanism that reduces an individual’s sensitivity to the effects of alcohol after repeated exposure. As individuals build tolerance to alcohol, they tend to consume larger amounts to achieve the desired level of intoxication (or the same level of drunkenness they previously experienced with lower amounts). This will result in excessive drinking, and they will experience withdrawal symptoms upon abstinence, which will encourage them to drink again and develop dependence. Tolerance is a form of homeostatic plasticity, which is the brain’s ability to maintain equilibrium upon alcohol exposure. Consumption of large doses of alcohol leads to central nervous system (CNS) depression, including increased activity of the inhibitory system, such as GABAergic neurons. This can trigger adaptive changes in the brain to restore overall brain activity levels. These adaptations are behaviorally reflected in the development of tolerance and dependence, making them essential components in the study of AUD [13,14,15].

To understand the mechanism of AUD and the development of tolerance, invertebrate model organisms such as *Drosophila melanogaster* and *Caenorhabditis elegans* have emerged as valuable tools. Their genetic tractability and conserved neural signaling pathways make them useful in investigating ethanol-induced tolerance and neuroplasticity at the molecular level. This literature review explores the insights gained from studying these phenomena in invertebrates, elucidating the intricate details of the molecular alterations caused by alcohol exposure/consumption, focusing on neurotransmission and synaptic mechanisms.

## 2. AUD and Neuroplasticity

**Figure 1:**
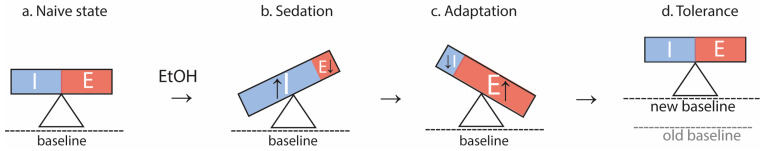
Dynamics of inhibitory (I) and excitatory (E) state of a neural circuit in response to ethanol (EtOH). Neuronal excitation and inhibition are balanced in the naive state (**a**). Exposure to ethanol alters this balance by increasing inhibition in the CNS, leading to sedation (**b**). To counteract the increased inhibition caused by ethanol, the CNS responds by increasing excitation (**c**). This adaptation leads to a new state where the balance between excitation and inhibition is achieved (**d**). This rebalanced state, achieved through the compensatory increase in excitation, is known as tolerance.

An essential aspect of understanding the progressive nature of AUD is how alcohol exposure causes alterations in brain structure and function, also referred to as neuroplasticity. Alcohol, a central nervous system depressant, has the ability to disrupt neuronal homeostasis by altering the balance between excitatory and inhibitory signaling within the brain [13,14]. These disruptions in neuronal balance are critical components of the development of both tolerance and dependence. Tolerance of prolonged alcohol use increases neuronal excitability as a compensatory mechanism to alcohol-induced CNS depression and is aimed at restoring equilibrium within the brain. This imbalance becomes particularly evident in conditions such as delirium tremens experienced during withdrawal. Individuals undergoing the withdrawal process experience severe discomfort, often accompanied by tremors and seizures, upon the discontinuation of alcohol consumption, all of which can be attributed to hyperexcitability of the CNS [16]. The heightened excitability of neurons reflects the brain’s adaptive process of acquired tolerance to counterbalance the CNS-depressant effects of alcohol (Figure 1).

## 3. Alcohol Tolerance—A Critical Endophenotype of AUD

While neuroplasticity is central to the development of AUD, the disorder’s complexity arises from its multifaceted etiology, influenced by a number of genetic, psychosocial, and environmental factors [17,18,19,20,21]. According to the Diagnostic and Statistical Manual of Mental Disorders (DSM-V), a diagnosis of AUD requires the presence of at least two of the 11 potential symptoms within a 12-month period. This can further be classified based on the severity depending on the number of symptoms presented by the patient; the presence of 2–3 and 4–5 criteria is diagnosed as a mild and moderate symptom, respectively, whereas the presence of 6 or more criteria can be presented as a severe form of AUD [22]. Because of its diverse clinical manifestation, it is important to explore the addiction’s endophenotypes, which are smaller, measurable components of a broader phenotype that enable us to bridge the gap between complex traits and the underlying genetic and biological mechanisms associated with AUD [23,24].

One of the risk factors for AUD is the initial sensitivity. Sensitivity refers to an individual’s naive response to the effect of alcohol. Some individuals are more sensitive and experience a heightened response at a lower dose. Others experience a reduced response to alcohol’s effects, requiring a larger dose to feel intoxicated, which can lead to escalated drinking and the development of tolerance. Tolerance is one of the major endophenotypes of AUD, which is defined as a reduced sensitivity to the intoxicating effect of alcohol following prior exposure, ultimately leading to AUD [25]. Tolerance is a form of neuroadaptive response in which an organism repeatedly exposed to alcohol develops resistance to its intoxicating effects [15]. Tolerance may result from more efficient removal of alcohol from the body, referred to as metabolic tolerance, or can also result from neural adaptations, referred to as functional tolerance. Functional tolerance can develop shortly after a single exposure to alcohol, known as acute tolerance, which can manifest within minutes of exposure. Alternatively, it may develop gradually over extended periods of time due to repeated or prolonged alcohol exposure, referred to as rapid or chronic tolerance. In humans, chronic alcohol use leads to tolerance, which can develop into both the pleasurable and aversive effects of ethanol [26]. Tolerance develops into the intoxicating effects of alcohol, such as motor imbalance or even becoming sedated. It also develops into the pleasurable effects of alcohol, such as feeling disinhibited and elated. Therefore, to replicate the feeling of elation, individuals need to drink more as tolerance develops to reach the same behavioral point of elation. This drives escalation, with dependence as a severe manifestation, where the Excitation/Inhibition (E/I) balance is so skewed that alcohol is needed on board [27]. The blood alcohol concentration (BAC) is a direct measure of the amount of alcohol present in a person’s bloodstream and can be used to assess both sensitivity and acute tolerance [28]. Individuals with higher sensitivity will rapidly show behavioral responses to the intoxicating effect as the BAC becomes higher, whereas individuals who develop acute tolerance will experience lower effects as they drink more, and BAC levels stay high [29]. Unlike acute tolerance, there is no efficient and direct way to measure rapid tolerance in humans (such as measuring naïve response to alcohol, followed by a second exposure to alcohol once alcohol from the first exposure has been metabolized). This review will focus on functional tolerance (pharmacodynamic tolerance) referred to as alcohol-induced alcohol tolerance.

## 4. Invertebrates as A Model System to Study AUD

Given the limited capacity for gaining mechanistic insights from human studies, the use of model organisms is essential in comprehending AUD and related behaviors. Invertebrates such as *Caenorhabditis elegans* (nematodes) and *Drosophila melanogaster* (fruit/vinegar flies) are valuable research models for investigating the roles of individual genes and proteins in neurotransmission, particularly those associated with alcohol-induced behaviors and neuroplasticity [30,31]. Other invertebrate models such as *Schmidtea mediterranea*, *Giradia tigrina* (planaria), *Apis mellifera* (honey bees), and *Orconectes rusticus* (crayfish) have also been used as promising models to study alcohol-related behaviors; however, they have been studied more sporadically and with lesser molecular and mechanistic amenability [32,33,34]. Therefore, this review will focus on the studies in *Drosophila* and *C. elegans*.

*C. elegans* serves as an excellent model organism due to its conserved neurobiological systems as well as the availability of numerous molecular and genetic tools, including a fully sequenced genome, thousands of genetic mutants, and the ability to manipulate gene expression through transgenic and RNAi techniques [35,36]. They also have a short life cycle and fast generation time (~3 days) allowing research at economy of time and scale compared to higher-level organisms. Microarray analysis has identified 230 genes to be affected by ethanol in *C. elegans* [37]. Furthermore, ~50 genes have been shown to influence alcohol-related behavioral responses in worms, and some of those genes orthologs have been implicated in alcohol use disorder in humans [38].

**Figure 2:**
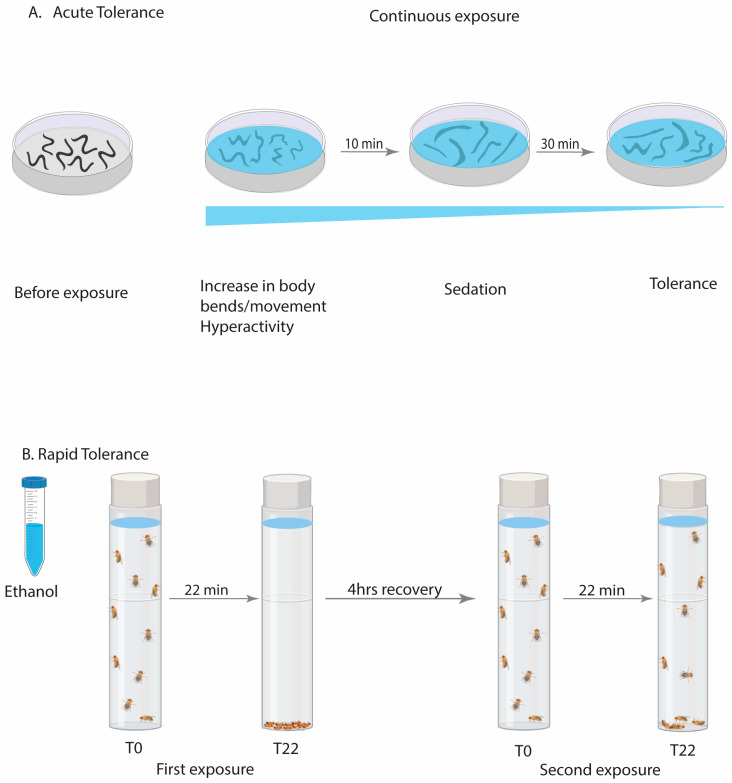
Different types of tolerance determined in invertebrates. (**A**) Schematic of development of acute functional tolerance in *C. elegans* during a single session of ethanol exposure. *C. elegans* respond to ethanol by first increasing their movement (or body bends) followed by progressive lack of coordination and eventual immobility within 10 min of exposure. After 30 min, they recover their locomotion speed despite the continued presence of ethanol on the agar plate, indicating the development of acute tolerance. (**B**) Schematic of rapid tolerance in *Drosophila*. The flies are exposed to ethanol vapor by pipetting it onto the vial plugs. After 22 min of exposure, all the flies become sedated. Flies are left to recover for 4 h, which allows the ethanol from the first exposure to be fully metabolized. When the flies are re-exposed to the same dose of ethanol, they take longer to be sedated than the first exposure, indicating the development of rapid tolerance.

*C. elegans* exhibit a range of dose-dependent behavioral responses to ethanol exposure that are similar to those observed in mammals. At a lower concentration, ethanol can lead to an increase in locomotor activity (hyperactivity) while a higher concentration of ethanol can lead to progressive changes in locomotion, ranging from incoordination to immobilization. These behavioral effects are reversible upon the removal of ethanol [39]. Furthermore, *C. elegans* develops acute tolerance to the depressive (or sedative) effects of ethanol (Figure 2A). This is demonstrated by their increased locomotor activity within 30 min of exposure despite the continuous presence of ethanol [40]. This indicates that they need more ethanol to reach the initial level of behavioral impairment after exposure, as their neurons have adapted to compensate for the effects of the alcohol. These effects occur when internal ethanol concentration reaches levels comparable to human intoxication [41,42]. The internal ethanol concentration of worms exposed to 400–500 mM ethanol for 22 min is comparable to 0.1% BAC [42]. Thus, *C. elegans* exhibits biphasic responses to ethanol, like mammals, which makes it a successful model for studying the molecular mechanisms underlying ethanol actions. These even include a model for experience-dependent preference, where pre-exposure to ethanol (for 4 h) induces adaptive changes that cause ethanol preference with a shift in behavior from avoidance to attraction [43].

Similarly, *Drosophila* exhibits a biphasic behavioral response to ethanol vapor, including an initial increase in locomotion followed by incoordination, loss of postural control, and eventual sedation and immobility similar to mammals. The concentrations of ethanol that stimulate locomotion in flies are similar to the levels in rodents and humans that induce disinhibition and feelings of euphoria. Similarly, the ethanol dose that causes incoordination and sedation in flies is also comparable to the effects observed in mammals [44]. The internal ethanol concentration at times of hyperactivity is about 20 mM (corresponding to a 0.09% BAC) and 45 mM at the time of sedation (corresponding to 0.21% BAC) [44]. Each of the behavioral responses can be determined using various assays, such as locomotor tracking and loss of righting test. These assays have evolved from using straightforward line-crossing assays to measure the fly’s speed for hyperactivity to video-tracking different behaviors [44,45,46], as well as using two-choice consumption assays to determine the flies’ experience-dependent alcohol consumption preference [47,48,49]. Meanwhile, the inebriometer provides a means to gauge both sedation levels and the impact on postural control. This is achieved by measuring the duration it takes for ethanol-exposed flies to lose postural control and elute out of a column filled with ethanol vapor [50]. Researchers also showed that flies develop rapid tolerance, indicating that they require a longer time to sedate upon a second exposure once all the ethanol from the initial exposure has completely metabolized (Figure 2B). Rapid tolerance can manifest in as little as two hours and can persist for at least 24 h and is a functional adaptation, which is not caused by altered ethanol metabolism [51,52]. Furthermore, upon chronic exposure, flies become dependent on ethanol and experience withdrawal symptoms upon removal of ethanol [53]. Thus, flies have been shown to develop tolerance, experience withdrawal, and show a preference for ethanol (they learn to like alcohol), making them another excellent model to study alcohol and associated behaviors.

Additionally, *Drosophila* has a short generation time (~2 weeks), a fully sequenced genome, a mapped central brain connectome, and molecular conservation. There are extensive genetic tools, such as Gal4/UAS transgene systems, that allow for a targeted gene expression to study gene function and behaviors in *Drosophila* [54]. This is a binary system consisting of Gal4, a yeast transcription factor that can be expressed in specific patterns, and UAS, an upstream activating system, a DNA sequence that contains the binding site for Gal4. When tissue-specific Gal4 binds to UAS, it promotes the transcription of genes downstream of UAS, allowing expression in a specific pattern (as driven by the Gal4). Other complementary binary systems, such as LexA/LexAop and QF/QUAS derived from bacteria and *Neurospora crassa*, respectively, have been used extensively for in vivo manipulations [54]. Similarly, the FLP/FRT system, derived from the yeast *Saccharomyces cerevisiae*, can be used for site-specific recombination and genetic manipulation [55]. In addition, the split Gal4 system, in which Gal4 is split into two fragments, a DNA binding domain (DBD) and the transcription activation domain (AD), enables intersectional expression patterns by producing a functional Gal4 protein only in cells expressing both AD and DBD [56,57]. Using this system, we can drive tissue-specific expressions of RNAi and CRISPR/Cas9 systems for conditional knockout and mutagenesis, respectively [58,59]. Using these available tools, researchers can activate, silence, or ablate specific targets (neurons/genes) to understand their role in mediating specific behaviors [60]. Furthermore, the use of Mosaic analysis with a repressible cell marker (MARCM) allows for tracing cell lineages and clonal analysis and can be used for understanding the development of the nervous system, as well as mapping neural circuits [54]. In addition, unbiased screens can also be used to identify different genes and pathways impacting alcohol phenotype [61,62]. Furthermore, the neurotransmitter systems in flies are evolutionarily conserved and serve analogous functions to those in mammals, making *Drosophila* a relevant model for studying these systems [63].

Thus, both *Drosophila* and *C. elegans* are useful and exhibit numerous alcohol-related phenotypes and behaviors similar to mammals. These alcohol-related behaviors/responses are also induced by similar ethanol doses/concentrations, proving that studying these models is relevant and useful. In addition, there is a high genetic conservation between invertebrates and mammals. Approximately 75% of human disease-associated genes have a fly ortholog [64]. Researchers have also shown a high level of homology (~83%) between *C. elegans* and human proteins [65]. Moreover, many genes implicated in AUD are also conserved and have been shown to have ethanol phenotype in both *C. elegans* and *D. melanogaster* [66]. But these invertebrate models have certain limitations: their nervous systems are not as complex as mammals. The anatomy and the neural circuitry of invertebrates and vertebrates are fundamentally different since the mammalian nervous system has more than 1,000,000 neurons, potentially reducing the translational relevance in modeling neuropsychiatric disorders [33]. In addition, invertebrates do not display the full range of behavioral diversity observed in mammals. Addictive behaviors, including the measurement of tolerance in invertebrate models, are performed via changes in locomotor activity on exposure. The behavioral repertoire in mammalian models is more complex, along with motor incoordination in response to intoxication, and various neurophysiological and imaging techniques have been employed to understand addiction [67,68]. However, the limitations shown by invertebrate models are balanced by the economy of scale and time since we can test many genes and their impact in a time span that would not be feasible in vertebrate systems.

## 5. Molecular Pathways Involved in Functional Tolerance Relating to Plasticity

### 5.1. Neurotransmitters and Peptides

#### 5.1.1. Octopamine

Octopamine is a biogenic monoamine that functions as a neurotransmitter and neuromodulator in invertebrates, including *Drosophila* and *C. elegans*. It is the invertebrate analog of norepinephrine, which signals via a G-protein coupled receptor to modulate numerous physiological and behavioral processes in *Drosophila*, including mating and courtship behavior, rhythmic behavior, and locomotion [69]. Octopaminergic signaling has been shown to play a significant role in the modulation of different ethanol responses in *Drosophila* such that the activation of octopaminergic signaling has been shown to increase attraction to ethanol odor [70] and the activation of a subset of octopaminergic neurons is sufficient to induce olfactory ethanol preference [71]. Scholz and colleagues show that flies that are incapable of synthesizing octopamine due to the lack of tyramine β-hydroxylase, an enzyme necessary for the synthesis of octopamine, show decreased tolerance to the sedative effect of ethanol [52]. The pharmacological depletion of norepinephrine in the brain prior to chronic ethanol exposure in mice prevented the development of tolerance to ethanol, highlighting the importance of an intact noradrenergic system for the development of tolerance in mammals [72]. The exact mechanism through which octopamine (or norepinephrine) influences ethanol response is not understood well, but it could involve interaction with other neurotransmitter systems. As a neuromodulator, octopamine may enhance or reduce neuronal excitability depending on the circuitry and neuronal population. This modulation can shift the overall balance of excitation and inhibition in a neural network, ultimately leading to changes in behavior.

#### 5.1.2. NPR-1

The neuropeptide receptor-1 (NPR-1) in *C. elegans* is a G-protein coupled receptor that plays a role in modulating various behaviors like locomotion, feeding, mating, and worms’ responses to different sensory and environmental cues. This neuropeptide receptor is known to be a part of a complex circuitry that integrates various signals (like food, oxygen, and pathogens) to coordinate behavioral outputs (like aggregation, locomotion, pathogen avoidance, and sleep) in *C. elegans* [73,74]. Npr-1 has been found to negatively regulate the development of acute tolerance to ethanol [40]. The *Drosophila* analog of NPR-1, neuropeptide F (NPF), and its receptor have also been found to modulate alcohol sensitivity. The overexpression of NPF causes sensitivity to ethanol, whereas reduced NPF expression has been linked to increased ethanol consumption, preference, and resistance to ethanol sedation in flies [75,76,77]. In mammals, the alteration of NPY signaling (mammalian ortholog) has been associated with changes in alcohol consumption, and abnormal or low NPY activity promotes increased drinking [78]. Although there is no evidence of the role of NPY signaling in alcohol-induced tolerance in flies and mammals, research shows that NPY signaling decreases baseline GABAergic transmission and reverses the alcohol-induced enhancement of inhibitory transmission in the central amygdala [79]. This highlights the importance of this neuropeptide signaling in alcohol-induced behaviors, as well as in maintaining the balance of excitatory and inhibitory signaling in the CNS. NPY signaling-modulated change in GABAergic transmission could be important in understanding the neuroadaptive changes leading to tolerance.

#### 5.1.3. GABA

Gamma-aminobutyric acid (GABA) is the primary inhibitory neurotransmitter in the CNS and is essential for maintaining the firing rate of a neuron through its action on ionotropic or metabotropic receptors. Large-dose ethanol exposure increases GABA signaling in the brain, leading to enhanced GABAergic inhibitory transmission. This increase in GABA signaling is known to contribute to the sedative effects of ethanol [80,81]. *Drosophila* ionotropic GABA_A_ receptor, with resistance to dieldrin (RDL), contributes to ethanol-induced locomotor stimulation, and the metabotropic GABA_B_ receptor has been shown to influence alcohol sensitivity and tolerance [82,83,84,85]. Dzitoyeva and colleagues showed that the administration of GABA_B_ agonist prior to ethanol exposure prevented the development of rapid ethanol tolerance and decreased ethanol sensitivity, and GABA_B_ antagonist increased resistance to the motor-impairing effect of alcohol in flies. Studies in *C. elegans* revealed the downregulation of genes associated with the GABA signaling pathway, including genes that encode glutamate decarboxylate (*unc-25*), GABA transporter (*unc-47*), and GABA receptor (*unc-49*), in response to ethanol [86]. Several studies have demonstrated that positive allosteric modulators of GABA_B_, as well as the GABA_B_ agonist baclofen, are effective in reducing alcohol-seeking and drinking behavior, and they have been used to treat withdrawal symptoms in mammals, including humans [87,88,89]. Baclofen can inhibit the development of rapid tolerance to ethanol in mice, while GABA_B_ antagonists can promote the development of tolerance in a dose-dependent manner [90]. This suggests that GABA_B_ signaling plays a significant role in the neuroadaptations that lead to the development of tolerance. One of the possible mechanisms for this adaptation could be a reduction in the number of receptors to compensate for the ethanol-induced activation of GABA receptors, which results in an increase in inhibition in the CNS, leading to ethanol-induced sedation.

### 5.2. Ion Channels

#### 5.2.1. NMDAR

N-methyl-D-aspartate receptors (NMDARs) are ionotropic receptors activated by the neurotransmitter glutamate and are essential for excitatory signaling in the CNS. NMDAR is one of the major targets of ethanol, as acute exposure to ethanol is known to inhibit NMDA-activated current while chronic exposure is known to increase the activity of NMDAR [91,92,93]. In *Drosophila*, a point mutation in NMDA receptor subunit 1 (NMDAR1) leads to a change in sensitivity to the effect of ethanol. F654A mutant flies exhibited increased sensitivity, while K558Q mutants showed decreased sensitivity to ethanol sedation without affecting tolerance [94]. Loss of function (LOF) mutant in *Drosophila Nmdar1* showed a decrease in the development of rapid tolerance to ethanol sedation [95]. Several studies have shown NMDAR activity to be associated with the development of acute tolerance to ethanol-induced inhibition in the mammalian brain [96,97]. The administration of NMDAR antagonists has been shown to block the development of rapid tolerance in mammals, highlighting the importance of functional NMDAR in adaptive response to alcohol [98,99,100]. Chronic alcohol exposure has been shown to increase the expression of the NMDAR subunits (NR1 and NR2) [93]. The increase in the number of receptors following ethanol exposure could reflect the brain’s adaptive process of acquired tolerance in order to compensate for the depressant effects of ethanol. This may be one of the mechanisms for the development of tolerance. NMDAR could also interact with synaptic proteins such as PSD95 and CASK to regulate ethanol tolerance (see below in Synaptic proteins).

#### 5.2.2. KCNQ

KCNQ is a family of voltage-gated potassium channels that play a crucial role in the regulation of the resting membrane potential and the control of neuronal excitability [101]. Ethanol has been shown to reduce the KCNQ current, and loss of *KCNQ* function in *Drosophila* increases sensitivity and tolerance to the sedative effects of ethanol [102]. Voltage-gated potassium channels like *KCNQ2*, *KCNQ3*, and *KCNQ5* have been shown to be associated with ethanol consumption and preference in rodents and humans [103,104]. The administration of potassium channel opener retigabine significantly decreases alcohol drinking in rodents and alleviates alcohol-induced anxiety during withdrawal [105,106,107]. Ethanol most likely alters neuronal excitability and affects neurotransmitter release via changes in KCNQ channel activity, leading to behavioral responses to ethanol like sensitivity and tolerance.

#### 5.2.3. BK Channel

The big potassium channel, also known as the slo1 channel, is a large conductance, calcium-, and voltage-activated potassium channel that plays a significant role in regulating neuronal excitability [108,109]. In *Drosophila*, the BK channels play a central role in the development of rapid tolerance to ethanol. Ethanol increases neuronal *slo-1* gene expression which leads to the development of rapid tolerance, while the deletion of *slo-1* results in loss of rapid tolerance [110,111]. Forward genetic screening revealed the BK channel to be one of the major targets of alcohol in *C. elegans*. Loss of function *slo-1* mutants were resistant to the effect of ethanol. The authors also demonstrated that ethanol activates the BK channel in vivo, which leads to the inhibition of neuronal activity, and the hyperactivation of the BK channel leads to behavior similar to ethanol intoxication [42]. Ethanol exposure was shown to downregulate the expression of *slo-1* in *C. elegans* [86]. *KCNMA1*, the mammalian ortholog of *slo-1*, has been associated with alcohol dependence in humans and other alcohol-related behaviors like sensitivity in rodents [103,112,113,114]. The role of the BK channel in alcohol-related behavior has been reviewed in detail [115,116,117,118]. In response to alcohol, the BK channel may directly modulate neuronal firing and excitation by regulating potassium conductance, which ultimately leads to changes in neurotransmitter release and subsequent behavioral changes associated with alcohol that lead to tolerance.

### 5.3. Synaptic Proteins

#### 5.3.1. Dlg1

The *Drosophila* gene *discs large 1* (*dlg1*), also referred to as the discs large homolog, encodes proteins DlgA and DlgS97, which are conserved orthologs of mammalian PSD-95 and SAP97 respectively. They are a member of the membrane-associated guanylate kinase (MAGUK) family of scaffolding proteins and play a key role in the organization of synapses, cell polarity, the composition of glutamate receptor subunits, and localization of calcium channels [119,120]. Loss of function mutants of *dlg1*, specifically encoding DlgS97 isoform, in *Drosophila* exhibit reduced tolerance to the sedative effects of alcohol. The authors also demonstrate that the deletion of *SAP97*, a mammalian homolog of dlgS97, leads to an inability to develop rapid tolerance to the sedative effects of ethanol in mice [95]. The role of *dlg1* has been associated with the regulation of NMDA receptor function at the synapse, which is a known target of alcohol [93,121,122]. The authors further show that the development of rapid tolerance may involve the interaction between DlgS97 and NMDAR subunit dNR1 [95]. This interaction may be important in the clustering and anchoring of glutamatergic receptors like NMDAR to the synapse, thereby influencing synaptic plasticity and the development of tolerance.

#### 5.3.2. CASK

CASK, also known as calcium/calmodulin-dependent serine protein kinase, is a scaffolding protein in the nervous system that plays an important role in synaptic function, neurotransmitter release, courtship behavior, and locomotion [123,124,125]. The LOF mutants of *CASK* display increased sensitivity and decreased ethanol tolerance in flies [95]. Although CASK has not been shown to be associated with alcohol response in mammals, it is involved in the regulation of CAMKII (calcium/calmodulin-dependent protein kinase II) via autophosphorylation, which has been implicated in the behavioral effects of alcohol [123,126,127,128]. Both CASK and CAMKII have been shown to form a complex with NMDAR to mediate synaptic plasticity. Therefore, the development of tolerance may involve NMDA-mediated synaptic plasticity and synaptic organization [129,130,131].

#### 5.3.3. Homer

Homer proteins are scaffolding proteins located within the postsynaptic membrane of excitatory synapses in the brain and are known to mediate the function, distribution, and trafficking of the glutamatergic receptors. It is essential for various neuronal processes, including synaptic plasticity, actin cytoskeleton remodeling, and intracellular calcium signaling [132]. Ethanol exposure decreases the expression of homer mRNA in *Drosophila*. LOF mutations of the *homer* gene also exhibit increased sensitivity and reduced rapid tolerance to the sedative effect of ethanol [133]. In mammals, *Homer2* has been shown to play a role in the behavioral and neurochemical response to alcohol. An increase in *Homer2* expression has been reported in rodent models of alcohol dependence and withdrawal [134]. Deletion of *Homer2* in mice increased sensitivity to the sedative effect of ethanol [135]. Homer protein regulates neuronal function mediating postsynaptic signaling and can alter glutamatergic signaling, bringing changes in the level of excitation in the brain and ultimately altering the E/I balance leading to ethanol-induced plasticity.

#### 5.3.4. Shibire

The *shibire* gene in *Drosophila* encodes a dynamin protein, a GTPase that plays a crucial role in endocytosis, synaptic vesicle recycling, and membrane trafficking [136]. It is required for the internalization of clathrin-coated vesicles at the plasma membrane, which is essential for various cellular processes, including neurotransmission [137]. The acquisition of rapid tolerance to ethanol in *Drosophila* requires the *shibire* genes, as demonstrated by the blocking of tolerance with the temperature-sensitive endogenous mutant allele of the gene [138]. Several proteomic studies show that the expression of dynamin-1 is significantly changed in the mammalian brain including humans after alcohol consumption, highlighting the importance of the functional protein in modulating alcohol response [139,140,141,142]. Dynamin-1 has also been shown to interact with the BK channel and other SNARE proteins to modulate vesicle release and recycling [143]. It is possible that *shibire* and its encoded protein dynamin are crucial for the acquisition of tolerance by promoting endocytosis and membrane cycling. Altering the activity of the protein can affect the efficacy of synaptic transmission and prevent neuroadaptation like the development of tolerance.

#### 5.3.5. Syntaxin 1A

Syntaxin 1A is encoded by the syntaxin gene (*stx1A*), and it belongs to a conserved family of SNARE (soluble N- ethylmaleimide-sensitive factor attachment receptor) proteins that play a role in synaptic exocytosis and ion channel regulation [144]. The syntaxin proteins interact with other synaptic vesicle proteins and aid in the docking and fusion of synaptic vesicles at the presynaptic active zone in the plasma membrane, facilitating neurotransmitter release [145]. Although alcohol does not alter the mRNA expression of *Syntaxin1A* (*syx1A*), the temperature-sensitive mutant allele of *syx1A* failed to develop rapid tolerance in *Drosophila* [138]. The direct role of *syntaxin* in alcohol tolerance has not been studied in *C. elegans.* However, a single point mutation (R39C) in *unc-18* [also referred to as syntaxin-binding protein 1 gene (*Stxbp1*)], which decreases syntaxin-binding, has been shown to increase alcohol sensitivity in a Rab-3-dependent manner [146]. Rab-3 is a small G-protein known to interact with synaptic vesicles at the active zone to regulate vesicle release. LOF mutants of *rab-3* show decreased sensitivity to the effect of alcohol in both *C. elegans* and mice [147]. A single amino-acid polymorphism (D216N) in *Munc18*, the mammalian ortholog for *unc-18*, has been associated with alcohol preference in mouse models [148]. The transgenic mutant for *unc-18* (D214N) in *C. elegans* reduces the sensitivity to both the stimulatory and depressive effects of alcohol [149]. In addition, Syntaxin 12 has also been found to be associated with alcohol preference in mice [150,151]. Thus, syntaxin and its interacting synaptic protein partners can regulate the release of neurotransmissions. Ethanol might change the activity of the exocytotic machinery to cause adaptation in the presynaptic active zone that leads to an alteration in neurotransmission, resulting in the imbalance of excitation and inhibition in the neural circuit to promote tolerance. Further research is required to understand the relationship between the presynaptic proteins *syntaxin*, *rab-3*, and *unc-18* and their role in alcohol-mediated responses.

#### 5.3.6. Synapsin

Synapsin is a conserved family of presynaptic proteins that play a role in the regulation of neurotransmitter release [152]. These neuronal phosphoproteins are bound to the cytoplasmic surface of the synaptic vesicles, and when phosphorylated by protein kinases (such as PKA or CAMKII), they release synaptic vesicles allowing them to move to the membrane and be released for neurotransmission [153]. Although there are contradictory results, Synapsin has been shown to be involved in the development of tolerance. Godenschwege and colleagues showed that the deletion of the *Synapsin* gene (*Syn*) in *Drosophila* enhances rapid tolerance to ethanol without affecting initial sensitivity [154]. However, Engel and colleagues showed that syn null flies are resistant to the effect of ethanol during first exposure and show reduced tolerance [155]. This would need further studies to decipher the exact mechanism in which synapsin is involved. This discrepancy in tolerance phenotype could be due to the initial resistance of the *syn* mutant and the experimental setup. Our lab recently showed that there are “primary” and “secondary” tolerance mutants and that initial resistance can impact the subsequent development of tolerance [156]. Additionally, ethanol exposure has decreased *Syn* expression in the *Drosophila* brain, and *Syn* is regulated by ethanol in a Sir2-dependent manner [histone deacetylases] [155]. In mammals, ethanol exposure increases the phosphorylation of synapsin in a PKA-dependent manner, highlighting the role of synapsin in presynaptic response to ethanol-induced inhibition of CNS [157]. Thus, synapsin regulates the number of synaptic vesicles available for neurotransmitter release, and ethanol might alter the activity of synapsin to cause presynaptic adaptation that increases neurotransmitter release and alters the neuronal balance. Sadanandappa and colleagues showed that when phosphorylated, synapsin promotes the mobilization and clustering of synaptic vesicles at the terminals, increasing the GABA transmission to balance the excitatory signal and promote short-term olfactory habituation [158]. Thus, this provides additional support to our argument.

#### 5.3.7. SEB-3

*SEB-3* encodes a GPCR closely related to mammalian corticotropin-releasing factor (CRF) receptors and is known to regulate stress response, locomotor activity state or arousal, and behavioral response to ethanol in *C. elegans* [159]. Seb-3 signaling is required for the development of ethanol preference, compulsive ethanol seeking, and tremors during ethanol withdrawal [159,160]. Loss of function mutants of *seb*-3 do not develop acute functional tolerance, whereas gain of function mutants show enhanced acute tolerance to ethanol [159]. CRF1 receptor has been implicated in the regulation of ethanol response in mammals. Studies have shown that crf1 receptor antagonists attenuate stress-induced increases in ethanol consumption and decrease binge-like ethanol drinking in mice and rats. Chronic consumption of ethanol dysregulates CRF signaling as shown by the increase in CRF release in the specific areas in the limbic system of alcohol-dependent rats during withdrawal [161,162]. Seb-3 or CRF receptor signaling may modulate neuronal excitability and neurotransmission via a range of G-protein-mediated intracellular signaling mechanisms to promote neuronal communication and influence ethanol-related behavior.

#### 5.3.8. GPRK2

G protein-coupled receptor kinase 2 (Gprk2) belongs to the family of kinases that plays a key role in the regulation of G protein-coupled receptors (GPCRs) and their activity. It is involved in receptor desensitization and internalization via G-protein phosphorylation leading to recruitment of β-arrestin and termination of the signal [163]. LOF mutation of *Gprk2* reduces rapid tolerance after a single intoxicating exposure, increases alcohol-induced hyperactivity, and reduces sensitivity to the sedative effects of ethanol [164]. Chronic ethanol consumption in rats is associated with an increase in the binding of mu-opioid receptors with G-protein receptor kinase 2 (Grk2) in the hippocampus and is associated with the desensitization of the opioid receptor following chronic consumption [165]. Chronic alcohol consumption has also been shown to increase *Grk2* expression in the nucleus accumbens shell and aid with the response to Kappa opioid receptor agonist nalfurafine to reduce excessive drinking in mice [166]. Thus, this highlights the role of GPCR in ethanol-induced response, specifically tolerance; neurons might adapt to ethanol by adjusting the activity of GPCR at the synapse to induce changes in behavior.

## 6. Conclusions

*C. elegans* and *Drosophila* have been widely used as model systems to study the mechanism of ethanol-induced tolerance. Studies have shown that the development of tolerance is regulated by various proteins and signaling molecules that can alter the balance between excitation or inhibition in the central nervous system. These include neurotransmitters and neuropeptides, ion channels, and synaptic proteins that are critical in the organization of synapses and can modulate synaptic plasticity by regulating neurotransmitter release and neuronal excitability (see Table 1 for a summary of various proteins and signaling molecules implicated in tolerance). The interplay of these components shapes the behavioral response to alcohol. Other genes and proteins, that are beyond the scope of this review, have been identified to have played a role in alcohol tolerance, including genes regulating alcohol metabolism (*ADH*), stress response (e.g., *hangover*, *jwa*), circadian rhythm (e.g., *tim*, *per*, *cyc*), histone deacetylase (HDAC), and learning and memory genes [167,168,169,170,171,172,173,174]. They may directly or indirectly be involved in the development and acquisition of tolerance while modulating neuronal plasticity. A comprehensive list of conserved genes involved in ethanol response in *C. elegans*, *Drosophila*, and humans has been reviewed [38].

In addition, alcohol causes epigenetic changes, which can influence the expression of alcohol-responsive genes and can lead to presynaptic adaptation, resulting in changes in neurotransmitter release in *Drosophila* [177,178], *C. elegans* [169,179,180,181], and mammals [86,182,183]. Furthermore, NPFs and their receptor play an important role in the transgenerational response to ethanol in *Drosophila*. Furthermore, researchers showed that maternal repression of NPF, likely via epigenetic changes, mediates transgenerational inheritance of ethanol preference in *Drosophila* offspring over multiple generations [184]. Flies exposed to alcohol during development exhibit several traits, including reduced viability, developmental delays, reduced brain and body sizes, and altered alcohol responses that phenocopy Fetal alcohol spectrum disorder (FASD) [185,186]. Guzman and colleagues showed that pre-fertilization alcohol exposure in *C. elegans* can induce transgenerational effects on alcohol sensitivity in the F3 generation [187]. Thus, parental exposure to alcohol can lead to altered gene expression in subsequent generations, most likely to prime the offspring for better tolerance; however, the mechanisms underlying the transgenerational effect of alcohol exposure are still unclear.

While research shows that alcohol causes structural and functional changes in the brain to promote tolerance to ethanol, the specific brain region or the neurocircuitry necessary for the development of tolerance is still unclear. In *C. elegans*, the GCY-35/GCY-36—TAX-2/TAX4 signaling pathway in oxygen-sensing neurons URX has been shown to be involved in ethanol tolerance [188]. Researchers showed that *Sir2* is required in the α/β lobes of the mushroom bodies (MB), the learning and memory centers of the *Drosophila* brain to promote ethanol sensitivity and the development of ethanol tolerance [155]. Additionally, Gprk2 and homer-mediated ethanol response in *Drosophila* have been mapped to specific regions in the ellipsoid body (EB) neurons, a brain region that has been linked to locomotion and sleep [133,164]. However, understanding the role of alcohol exposure on other anatomical structures might be important as well, and alcohol-associated changes in the URX, MB, and EB still need further research. In addition, understanding the relationship between initial sensitivity/resistance and tolerance could provide us with a better understanding of “primary” tolerance mutants in *Drosophila* and their roles in the development of alcohol tolerance.

Although much of the research on alcohol-induced tolerance in animal models focuses on motor tolerance (tolerance to sedation), adaptation to alcohol is evident in various physiological systems, such as mammalian thermoregulation and worm egg-laying [42,189,190,191]. Motor tolerance may not fully capture the complexities of addiction or AUD; it is, therefore, crucial to explore other forms of tolerance to comprehensively understand addiction. One such form of tolerance, which is a major driving force that leads to addiction and relapse in humans, is hedonic tolerance. Hedonic tolerance refers to the neurobiological adaptation that leads to a reduction in the positive and pleasurable effects that occur with repeated exposure to a substance such as alcohol and an increase in the negative effect during its abstinence [15]. However, the molecular mechanisms underlying this process are still unclear. It is unclear whether *Drosophila* or *C. elegans* can experience hedonic tolerance; therefore, establishing models for alcohol-induced hedonic tolerance in these invertebrates could offer us a better genetic and molecular understanding of these adaptive mechanisms and addiction in general.

In addition to invertebrates, several rodent models have demonstrated that chronic alcohol exposure induces neuroplastic changes in the brain regions, specifically in the striatum and the bed nucleus of the stria terminalis (BNST). The authors further discuss that alcohol dysregulates glutamatergic, GABAergic, and neuropeptidergic signaling, similar to the invertebrate models [192]. Furthermore, GABA receptor agonists (Baclofen), GABA analog (Gabapentin), and NMDAR agonists (Acamprosate) have been used as medications to treat alcohol use disorder in clinical studies [193]. Additionally, chronic alcohol exposure decreases BDNF levels in various brain regions and has been associated with increased alcohol drinking and the development of tolerance [194,195]. Several other neurotrophins, like growth factors, have been shown to affect alcohol use in mammals, but not much has been studied in invertebrate models [196]. The role of neurotrophins in alcohol tolerance and dependence needs further research. In addition, the potential therapeutics for tolerance and dependence need to be investigated more. Drugs targeting epigenetic as well as synaptic regulators could provide new avenues for therapeutics for AUD.

Overall, the molecular mechanism underlying ethanol-induced plasticity in the context of the development of tolerance involves adaptation to different targets of alcohol that alters the balance in excitation and inhibition in the CNS. Further research is needed to fully understand these mechanisms and develop effective treatments for alcohol addiction.

## Figures and Tables

**Table 1:** Molecular pathways involved in functional alcohol tolerance relating to plasticity in invertebrate models and their associated mammalian phenotype.

Name	Invertebrate Model	Gene	Gene Function	Mutant	AUD-Related Behavior in Invertebrates	Assay	Dose	Phenotype in Mammals	References
Octopamine	Fly	*tyramine β-hydroxylase (TβH)*	Rate-limiting enzyme for octopamine synthesis.	LOF mutant [*TβHnM18*]	LOF mutants exhibit reduced rapid tolerance.	Inebriometer	50/45 ethanol/air	Norepinephrine depletion in the brain before exposure prevents the development of tolerance in mice.	[52,72]
Neuropeptide Receptor	Worm	*npr-1*	Neuropeptide Y-like receptor.	LOF mutant [*npr-1*]	LOF mutants show increased acute functional tolerance (AFT).	Rate of locomotion	500 mM ethanol exposure for 10, 30, or 50 min	NPY-deficient mice show increased alcohol consumption, are less sensitive to the effects of alcohol, and make a quick recovery after alcohol sedation. NPY overexpression shows low preference for alcohol and is more sensitive to the sedative effects of alcohol.	[40,77,175]
	Fly	*NPF/NPFR*	Neuropeptide F receptor.		NPY-deficient flies show decreased alcohol sensitivity, whereas overexpression leads to increased sensitivity to the effects of alcohol.	Time to sedation [Ethanol pipetted in Kim wipes and placed at the bottom of a fly bottle].	10%, 31%, 54%, or 100% ethanol vapor until complete sedation		
GABA	Worm	*unc-25*, *unc-49*, *unc-47*	Synthesis enzyme for GABA (glutamate decarboxylate), GABA receptor, and GABA transporter, respectively.		Genes that encode components of GABA signaling become downregulated after alcohol exposure.	Microarray	400 mM ethanol exposure	Acute alcohol exposure increases GABA_A_ receptor activity, resulting in increased neuronal inhibition and sedation. GABA agonist Baclofen has been shown to reduce alcohol consumption and craving and inhibit the development of rapid tolerance to ethanol in mice, while GABA_B_ antagonists facilitate rapid tolerance in a dose-dependent manner.	[82,85,86,87,90,176]
	Fly				GABAA contributes to ethanol-induced locomotor stimulation. Administration of GABA_B_ agonists decreased sensitivity and increased tolerance, while GABA_B_ antagonists increased alcohol sensitivity.	Booz-o-mat: Pharmacological administration followed by measurement of time to sedation	60% saturated ethanol vapor; 100% ethanol for 24 min		
NMDAR	Fly	*nmdar1*	NMDA receptor 1.	F654A and K558Q; LOF mutant	Two different point mutations in *NMDAR1* showed altered sensitivity to alcohol sedation, while loss of function mutation reduced rapid tolerance.	Inebriator; inebriometer	100% ethanol heated to 65 °C and a flow of 15 mL/min; 60/40 ethanol vapor/humidified air for 30 min	Administration of NMDAR antagonists has been shown to block the development of rapid tolerance in mammals. Chronic alcohol exposure has been shown to increase the expression of the NMDAR subunits (NR1 and NR2).	[93,94,95,99,100]
KCNQ	Fly	*KCNQ*	Voltage-gated potassium channel.	Null mutant [*KCNQ*^186^]	Ethanol has been shown to reduce KCNQ current and loss of *KCNQ* function in *Drosophila* increases sensitivity and tolerance to the sedative effects of ethanol.	Time to sedation [Ethanol pipetted in Kim wipes and placed at the bottom of a fly bottle].	40% ethanol for 90 min	*KCNQ2*, *KCNQ3*, and *KCNQ5* have been associated with ethanol consumption and preference in rodents and humans. Administration of potassium channel opener retigabine significantly decreases alcohol drinking in rodents and alleviates alcohol-induced anxiety during withdrawal.	[102,103,104,105,106]
BK channel	Worm	*slo-1* (*slowpoke*)	Big potassium channel.	LOF mutant [*slo-1*], *eg7*, *eg73*, *eg24*, and *eg142*; gain of function mutants [*ky389gf*, *ky399gf*]	BK channel is one of the major targets of alcohol. Loss of function *slo-1* mutants were resistant to the effect of ethanol. Ethanol activates the BK channel in vivo, leading to inhibition of neuronal activity, and hyperactivation of the BK channel leads to behavior similar to ethanol intoxication. Ethanol exposure downregulates *slo-1* expression.	Forward genetics, microarray; locomotor, egg-laying; Electrophysiology (whole-cell current recording-patch clamp).	400 mM ethanol exposure for 20 min	The mammalian ortholog of *slo-1*, *KCNMA1*, is associated with alcohol dependence in humans and other alcohol-related behaviors like sensitivity in rodents.	[42,86,103,110,111,112,114,116]
	Fly	*slo-1* (*slowpoke*)	Big potassium channel.	Null mutant [*slo^4^*]; *ash2^18^* (neuronal deletion of *slo*)	Ethanol increases neuronal *slo-1* gene expression, which leads to the development of rapid tolerance, while deletion of *slo-1* results in loss of rapid tolerance. Artificial induction of *slowpoke* makes flies more resistant to the effect of ethanol.	Inebriator	100% ethanol heated to 65 °C at flow of 15 mL/min		
Dlg1	Fly	*discs large 1* (*dlg1*)	Member of the membrane-associated guanylate kinase (MAGUK) family of scaffolding proteins.	LOF mutants [*dlg1*]; *dlg^NP1102^*, *dlg^NP768^*	LOF mutants show decreased tolerance to the sedative effects of alcohol.	Inebriometer	60/40 ethanol vapor/humidified air for 30 min	Deletion of *SAP97*, a mammalian homolog of *dlgS97*, leads to an inability to develop rapid tolerance to the sedative effects of ethanol in mice.	[95]
CASK (Calcium/calmodulin-dependent serine kinase)	Fly	*CASK*	Scaffolding protein (also known as Caki or Camguk).	LOF mutant [*CASK*]; *CASK^P18^*, *CASK^P46^*	LOF mutants of *CASK* display increased sensitivity and decreased ethanol tolerance.	Inebriometer	60/40 ethanol vapor/humidified air for 30 min		[95]
Homer	Fly	*homer*	Neuronal protein that regulates metabotropic glutamate receptor function.	null mutant [*homer^R102^*]	Ethanol exposure decreases the expression of homer mRNA in *Drosophila*. *LOF* mutants of *homer* exhibit increased sensitivity and reduced rapid tolerance to the sedative effect of ethanol.	Microarray, ethanol sensitivity, and tolerance assay	56% ethanol at a flow of 160 mL/min for 50 min for sedation; 50–70% at a flow of 130 mL/min for 40 min for tolerance	An increase in *Homer2* expression has been reported in rodent models of alcohol dependence and withdrawal.	[133,134,135]
Shibire	Fly	*shibire*	Encodes the GTPase dynamin which enables actin-binding activity and synaptic vesicle recycling	Temperature-sensitive mutant allele [*shi^ts1^*, *shi^ts2^*].	Temperature-sensitive endogenous mutation in shire prevents the development of tolerance.	Inebriator	100% ethanol heated to 65 °C at flow of 15 mL/min	The expression of *dynamin-1* is significantly changed in the mammalian brain including humans after alcohol consumption.	[138,139,140,141,142]
Syntaxin 1A	Fly	*syntaxin 1A*	part of the SNARE protein complex and is required for vesicle fusion.	Temperature-sensitive mutant allele [*Syx1A*^3−69^].	The temperature-sensitive mutant allele of *syx1A* failed to develop rapid tolerance.	Inebriator	100% ethanol heated to 65 °C at flow of 15 mL/min	*Syntaxin 12* has also been found to be associated with alcohol preference in mice.	[138,150,151]
Synapsin	Fly	*synapsin*	Neuronal phosphoprotein involved in the regulation of neurotransmitter release.	null mutant [*SAP^97^*]	One study showed that deletion of the *Synapsin* gene (*Syn*) enhances rapid tolerance to ethanol without affecting initial sensitivity, while the other showed that the flies are resistant to the effect of ethanol during first exposure and showed reduced tolerance. Exposure to ethanol decreases *Syn* expression in the brain, and the expression is regulated by ethanol in a Sir2-dependent manner [histone deacetylases].	Time to ST50 and tolerance; Booz-o-mat	50% ethanol vapor in perforated 50mL falcon tube; 85/65 ethanol vapor/air.	Ethanol exposure increases the phosphorylation of synapsin in a PKA-dependent manner.	[154,155,157]
SEB-3	Worm	*seb-3*	Encodes a corticotropin-releasing factor (CRF) receptor-like GPCR and is involved in stress response.	LOF mutant [*seb-3* (*tm1848*)], GOF mutant [*eg696*]	*Seb-3* signaling is required for the development of ethanol preference, compulsive ethanol seeking, and tremors during ethanol withdrawal. Loss of function mutants of seb-3 do not develop acute functional tolerance, whereas gain of function mutants show enhanced acute tolerance to ethanol.	Rate of locomotion	500 mM ethanol exposure for 10, 30, or 50 min	Studies have shown that crf1 receptor antagonists attenuate stress-induced increases in ethanol consumption and decrease binge-like ethanol drinking in mice and rats. Chronic consumption of ethanol increases CRF release in the specific areas in the limbic system of alcohol-dependent rats during withdrawal.	[159,160,161,162]
GPRK2	Fly	*GPRK2*	Encodes a member of the G protein-coupled receptor kinase family which modulates GPCR signaling activity.	LOF mutant [*gprk2^KO^*, *gprk2^del1^*]	LOF mutation of *Gprk2* reduces rapid tolerance, increases alcohol-induced hyperactivity, and reduces sensitivity to the sedative effects of ethanol.	Loss of righting reflex (LORR)	EtOH/water vapor (1:1) at a flow rate of 250 mL/min	Chronic ethanol consumption in rats is associated with an increase in the binding of mu-opioid receptors with G-protein receptor kinase 2 (Grk2) in the hippocampus and is associated with the desensitization of the opioid receptor following chronic consumption. Chronic alcohol consumption increases Grk2 expression in the nucleus accumbens shell to reduce excessive drinking in mice.	[164,165,166]

Columns are as follows: names of proteins, which invertebrate model is the evidence from, the gene symbol, a brief description of the function of the gene, the mutant used for the studies, AUD-related phenotypes in the invertebrate model, the assays used, the dose of alcohol used for the studies, the evidence from mammalian studies implicating the gene in some aspect of AUD, and the citations.

## Data Availability

Not applicable.
